# Curcumin and Metformin Infinite Coordination Polymer Nanoparticles for Combined Therapy of Diabetic Mice via Intraperitoneal Injections

**DOI:** 10.3390/jfb15120388

**Published:** 2024-12-21

**Authors:** Siwei Sun, Xinyi Hou, Ke Li, Chenqi Huang, Yu Rong, Jiao Bi, Xueping Li, Daocheng Wu

**Affiliations:** 1 Institute of Basic and Translational Medicine, Xi’an Medical University, Xi’an 710021, China; bs1808008@sust.edu.cn (S.S.);; 2 Xi’an Key Laboratory for Prevention and Treatment of Common Aging Diseases, Translational and Research Centre for Prevention and Therapy of Chronic Disease, Xi’an Medical University, Xi’an 710021, China; 3 School of Pharmacy, Xi’an Medical University, Xi’an 710021, China; 4 Key Laboratory of Biomedical Information Engineering of the Ministry of Education, School of Life Science and Technology, Xi’an Jiaotong University, Xi’an 710049, China; 5 School of Clinical Medicine, Xi’an Medical University, Xi’an 710021, China

**Keywords:** infinite coordination polymer, nanoparticles, metformin, curcumin, diabetes, combination therapy

## Abstract

Metformin (Met) is one of the most commonly prescribed first-line drugs for diabetes treatment. However, it has several issues, including low bioavailability, therapeutic platform, and side effects at high doses. In order to improve the therapeutic efficiency of Met, this study proposes a strategy of using Met and curcumin (Cur) to prepare Cur-Zn(II)-Met infinite coordination polymer nanoparticles (CM ICP NPs), and combining this with intraperitoneal injections, for the treatment of diabetic mice. Fourier transform infrared (FTIR) spectroscopy, X-ray photoelectron spectroscopy (XPS), transmission electron microscopy (TEM), nanoparticle analysis, cytotoxicity experiments, and mice experiments were used to investigate structure, properties, and application effects. The results showed that CM ICP NPs exhibit a high drug encapsulation rate (100%), good stability, and an absence of in vivo and in vitro toxicity. The blood glucose level of diabetic mice after treatment was reduced to 6.7 ± 0.65 mmol/L at the seventh week. In terms of therapeutic mechanism, it appears that Met and Cur can synergistically regulate blood glucose in mice from multiple paths. This study provides a promising method for the treatment of diabetes using Met and other drugs.

## 1. Introduction

Diabetes is one of the leading causes of death, second only to cardiovascular disease and cancer [1]. With the increase in high-calorie diets and unhealthy lifestyles, such as being sedentary, the number of diabetic patients worldwide has increased dramatically. Currently, there are over 400 million people with diabetes worldwide, and this number is expected to reach 700 million by 2030, accounting for 10.9% of the global population [2,3]. Due to its complex pathogenesis, involving multiple mechanisms such as insulin resistance and β-cell dysfunction, and its chronic nature, patients often require long-term use of hypoglycemic drugs to control their blood glucose levels [4]. Metformin (Met) is a commonly prescribed first-line drug for the treatment of diabetes, with a history of widespread use for over 40 years [5]. However, its oral bioavailability is only 50% to 60% [6]. Ordinary tablets are absorbed through the small intestine and have a plasma half-life of 1.5–5.0 h. It is circulated unbound in plasma and cleared, unchanged, by the kidneys [7]. The biphasic technique is used to reduce the loss of Met during circulation [8]. As the disease progresses, it often becomes necessary to continuously increase the dosage. When the daily oral dosage exceeds 2000 mg, a plateau period of hypoglycemic effect may occur [9]. Additionally, accumulation of the drug in the intestine can lead to serious gastrointestinal diseases, lactic acidosis, poor absorption of vitamin B12, and other side effects [10]. Furthermore, individual differences may result in some patients having a lower sensitivity to Met, leading to unsatisfactory hypoglycemic effects when used alone [11,12]. Therefore, improving the utilization of the drug and exploring alternative treatment pathways have become crucial in enhancing the efficacy of Met.

In recent years, the combination of available anti-diabetes drugs and traditional Chinese medicine (phytochemicals with pharmacological properties) has emerged as a significant treatment strategy for hyperglycemia and other diabetes-related conditions [13,14,15]. Curcumin (Cur) is a kind of natural effective ingredient with a β-diketone structure, extracted from the natural medicine Curcuma turmeric, turmeric, and other ginger plants. Cur can play an anti-diabetes role by regulating oxidative stress, inflammatory reaction, pancreatic islet cell function, and more [16]. A study found that Cur is 400 times more effective than metformin (Met) in activating AMPK enzymes (AMPK activation is the “therapeutic target” of diabetes) [17]. In addition, Cur can also mediate FGF23 to affect the blood glucose of diabetes patients through calcium and phosphorus regulation [18,19]. Combining Cur and Met can provide multiple benefits in treating diabetes. Hassan et al. [20] used Cur silver nanoparticles to enhance the efficacy of Met. Roxo et al. [21] used Cur and Met to reduce blood sugar and lipids in diabetic rats. However, combination therapy often involves direct fractional application or nanotechnology encapsulation, and still acts independently when entering the body for absorption and metabolism, resulting in low drug utilization, poor compatibility, mutual influence of action spaces, and poor stability [22,23,24]. In recent years, there has been rapid development in the field of infinite coordination polymer (ICP) drug delivery systems. These systems have the ability to simultaneously possess multiple drug characteristics, achieve multi-channel combined effects of different drugs, enhance drug efficacy, reduce adverse reactions, expand the scope of applicability, and reduce drug dosage [25,26,27]. ICPs are typically supramolecular polymer amorphous nanomaterials formed by the repeated coordination and self-assembly of organic ligands and inorganic metal ions [28]. Due to their high cutting ability, flexible selection of raw materials, controllable material size, and ease of deeper processing, they are widely used in drug delivery systems [29]. Our research group [30] formed ICPs by coordination with doxorubicin, gossypol, and Fe^3+^, which achieved the targeted delivery and graded treatment of various drugs to tumors. Huang et al. [31] constructed a novel radiosensitizer based on ICPs using hemoglobin, disodium 5′-guanylate, and Gd^3+^ to simultaneously perform X-ray deposition and glutathione depletion, promoting radiation-induced immunogenic cell death. ICPs have the ability to modify the interaction between metal ions and ligands as needed. Through a simple “one-step” reaction, multifunctional complexes with adjustable properties can be obtained. These composite ICPs have the capability to read data from multiple channels and utilize the stimulus response mechanism of binding bonds to the external environment. This allows for the creation of novel targeted functional drugs with ultra-small size and high dispersibility, enabling precise drug delivery and synergistic enhancement [32,33,34]. ICPs provide a feasible pathway for the combined delivery of Cur and Met [29]. In addition, injection administration can avoid first pass effects in the gastrointestinal tract or liver, and has the characteristics of low dosage, fast response, and minimal side effects [35]. Hyldbakk et al. [36] also used intraperitoneal injection to control the delivery pathway of cabozantine in mice. This also has advantages in the precise delivery of low-dose drugs.

**Figure 1:**
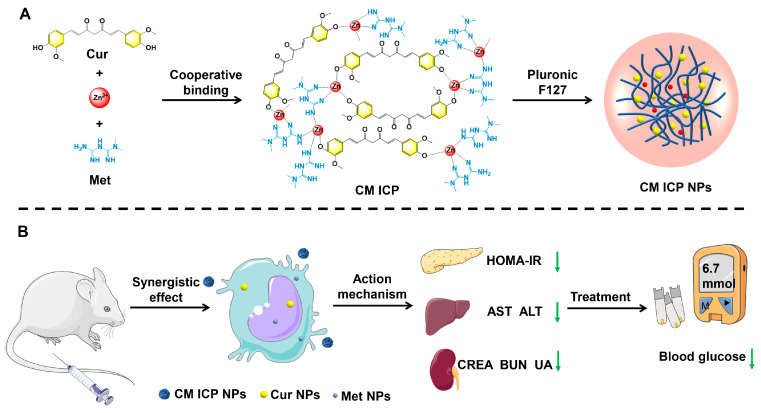
Synthetic route of CM ICP NPs (**A**) and their application in diabetes mice (**B**). (Curcumin, Cur; Metformin, Met; infinite coordination polymer, CM ICP; infinite coordination polymer nanoparticles, CM ICP NPs; Curcumin nanoparticles, Cur NPs; Metformin nanoparticles, Met NPs; insulin resistance index, HOMA-IR; aspartate aminotransferase, AST; alanine aminotransferase, ALT; creatinine, CREA; blood urea nitrogen, BUN; uric acid, UA; The green downward arrow indicates a decrease in the level of the indicator).

Therefore, this research is the first to apply infinite coordination technology in the preparation of diabetes combination drugs. Through a simple one-step reaction, Met and Cur are coordinated with Zn^2+^ to form a closely linked infinite coordination polymer (CM ICP), which is enveloped by Pluronic F127 to prepare infinite coordination polymer nanoparticles (CM ICP NPs). At the same time, intraperitoneal injection is used. It aims to build solutions to the problems of low bioavailability, therapeutic platform, and side effects at high doses that are commonly encountered during the medication process of Met. The therapeutic effect on diabetic mice was evaluated by measuring the sample structure, in vitro release performance, in vivo and in vitro toxicity of different treatments, and hypoglycemic performance. Additionally, the mechanism of action was further revealed by examining its effects on insulin resistance, lipid metabolism, and liver and kidney function (Figure 1). This study introduces a promising advancement in combination therapy for diabetes using coordination polymer dual-drug nanoparticles. This method provides synergistic therapeutic benefits, effectively addressing issues of drug utilization, compatibility, and stability in diabetes management.

## 2. Materials and Methods

### 2.1. Materials

Metformin hydrochloride (Met·HCl, purity > 97%), curcumin (Cur, purity > 98%), Zinc(II) acetylacetonate (Zn(C_5_H_7_O_2_)_2_), and trihydroxymethamine (Tris) were purchased from Shanghai Macklin Biochemical Technology Co., Ltd. (Shanghai, China). Sodium chloride, Triton X-100, Pluronic F127, methanol, ethanol, and dimethyl sulfoxide (DMSO) were obtained from Shanghai Aladdin Biochemical Technology Co., Ltd. (Shanghai, China). Normal Cell Line 3T3 (Mouse Embryonic Fibroblasts), RSC96 (Rat Schwann Cells), HT22 (Mouse Hippocampal Neuron Cell Line), and Sheep Erythrocyte (6%) were provided by Servicebio Biological Technology Co., Ltd. (Wuhan, China). Beyotime Biological Technology Co., Ltd. (Shanghai, China) provided Dulbecco’s modified Eagle’s medium (DMEM) and a CCK-8 reagent kit. All in vivo tests were authorized by the Biomedical Research Ethics Committee of Xi’an Jiaotong University, and conducted according to the Guidelines for Use and Care at Xi’an Jiaotong University.

### 2.2. Preparation of CM ICP NPs

Met (72.8 mg) and Cur (7.28 mg) were dissolved in 1820 μL. These two solutions were combined and sonicated at 50 kHz for 10 min to prepare Solution A. Subsequently, 100 μL of a Zn(C_5_H_7_O_2_)_2_ (4.368 mg) ethanol solution (10 mg/mL) was added dropwise to Solution A at a rate of 100 drops per minute. After the addition was complete, the mixture was stirred for an additional 10 min at 600 rpm, resulting in Solution B. Solution B was added to a mixture of Pluronic F127 (14.56 mg) and Tris-alkaline buffer solution in 80 μL DMSO, followed by stirring for 30 min at 1800 rpm, and maintaining a pH of 7.4, to obtain CM ICP NPs. The final product concentrations were adjusted to 3.64 mg/mL for Cur and 36.4 mg/mL for Met.

### 2.3. Chemical Structural of CM ICP NPs

Ultraviolet-visible absorption spectroscopy (UV-Vis) was conducted using a Lambda 950 spectrophotometer (Perkin Elmer, New York, NY, USA), with a scanning range of 200 to 800 nm. The functional groups of the samples were analyzed using an FTIR spectrometer (Bruker Optik GmbH, Ettlingen, Germany) with a scanning range of 400–4000 cm^−1^. X-ray photoelectron spectroscopy (XPS) was performed using an AXIS SUPRA instrument (Kratos, Manchester, UK), equipped with a water-cooled, focused monochromatic Al Kα radiation source. The Zn2p, N1s, C1s, and O1s peaks of the sample were analyzed, and data processing was carried out using Avantage software (version 5.9). The C1s energy was calibrated based on a binding energy of 284.8 eV. X-ray diffraction (XRD) analysis was performed using a D/max 2200PC instrument (Rigaku, Tokyo, Japan), with a scanning speed of 2°/min and a scanning angle range of 25° to 60°.

### 2.4. Characterization of Morphology and Release

The morphology, particle size, elemental distribution, and zeta potential of the CM ICP NP samples were analyzed using transmission electron microscopy (TEM, JEM-F200, JEOL Japan Electronics Co., Ltd., Tokyo, Japan), an energy dispersive spectroscopy (EDS) system (FEI Tecnai F20, FEI, Hillsboro, NJ, USA), and dynamic light scattering (DLS) with a Malvern Nano-ZS90 instrument (Malvern Instruments, Birmingham, UK). The particle size stability of the CM ICP NPs was monitored over a period of 40 days.

Additionally, the drug release profiles of the CM ICP NPs were evaluated. The CM ICP NPs were encapsulated in dialysis bags with a cut-off molecular weight of 3.5 kDa, and then immersed in PBS solutions with pH values of 7.4, 6.5, and 5.2, respectively. The dialysis process was conducted for 65 h at 25 °C by magnetic stirring with 30 rpm. The dialysate was collected at different time points, and the concentration of Cur was measured by UV-Vis at λ = 427 nm.

### 2.5. Animal Experiments

We confirm that we adhered to the guidelines for the use of animals in bioscience research as outlined in China. All ethical considerations were taken into account according to the appropriate standards in China. All animal experiments were approved by the Laboratory Animal Ethics Committee of Xi’an Medical University, with approval number XYLS2021209.

All animals were of specific-pathogen-free grade. 14 days of health observation and adaptive feeding were conducted before the experiment, while environmental conditions and care that are consistent with those for future feeding were provided. Furthermore, we employed an appropriate experimental design to minimize the harm and number of animals as much as possible, while ensuring the repeatability of our treatments within a margin of error. During the experiment, scientific and proficient operating techniques and prompt pacification were employed.

### 2.6. Toxicity In Vitro and In Vivo

#### 2.6.1. In Vitro Toxicity Tests (CCK-8 Experiment)

Since CM ICP NPs are administered with intraperitoneal injections, and Met and Cur differ greatly in water, the same dose of intraperitoneal injections for the drug as for the CM ICP NPs could not be achieved. Therefore, we made Pluronic F127 nanoparticles loaded with Met and Cur (curcumin nanoparticles: Cur NPs, and metformin nanoparticles: Met NPs), as well as mixed Cur and Met nanoparticles (Cur–Met NPs), thus using the same dose of Met NP and Cur NP intraperitoneal injections as a control. Future biological experiments will be carried out according to this. The preparations of Cur NPs, Met NPs and Cur–Met NPs are provided in the Supplementary Information.

Cell line mouse embryonic fibroblasts (3T3), rat Schwann cells (RSC96), and mouse hippocampal neurons (HT22) were seeded into a 96-well plate at a density of 8000 cells per well. Subsequently, CM ICP NPs, Cur NPs, and Met NPs were added at concentrations of 1 μg/mL, 3 μg/mL, and 9 μg/mL, respectively. The cells were incubated at 37 °C in a 5% CO_2_ environment. Once the cells in the control wells reached 90% confluency, the culture medium was replaced with a colorless medium containing 10% CCK-8 reagent. After a 2 h incubation period, the absorbance of each well was measured at 450 nm, using an enzyme-linked immunosorbent assay (ELISA) reader (Multiskan SkyHigh, Thermo Fisher Scientific, Waltham, MA, USA), to assess cell viability.

#### 2.6.2. In Vivo Hemolysis Test

CM ICP NPs, Cur NPs, Met NPs, Saline, and Triton X-100 were added to Eppendorf tubes containing a 2% red blood cell suspension (6% Sheep Erythrocyte was diluted with saline solution), with each component making up 15% of the total solution volume. The mixtures were incubated at 37 °C for 2 h, after which the sedimentation of red blood cells was observed. The absorbance of the supernatant was measured at 490 nm using an ELISA reader, and the hemolysis rate was calculated.

#### 2.6.3. In Vivo Toxicity Test

C57 mice (balanced for sex, approximately 25 g each) were randomly divided into three groups (*n* = 13 per group): a CM ICP NPs group, a mixed Cur–Met NPs group (the preparation of Cur–Met NPs is provided in the Supplementary Information), and a Saline group. The mice were intraperitoneally injected with the corresponding treatments, with all dosages adjusted to 30 mg/kg for Cur and 300 mg/kg for Met. The mice were prohibited from eating and drinking for 6 h before injection, and allowed to resume after 2 h. At the 24th hour, three individuals were randomly selected from each group to collected their eyeball blood. The mice were then monitored daily for their behavioral habits and mental state, including the conditions of insanity, messy hair, difficulty breathing, cachexia, astasia, fighting, and coma, over a 14-day period. Their survival rate was calculated. On the 14th day, the surviving mice were euthanized. Three mice were randomly selected from each group for eyeball blood collection. Blood samples were used to assess key indicators of liver function, including aspartate aminotransferase (AST), alanine aminotransferase (ALT), and total bilirubin (TBIL), as well as kidney function markers such as creatinine (CREA), blood urea nitrogen (BUN), and uric acid (UA). Their livers, kidneys, hearts, lungs, and spleens were taken for hematoxylin and eosin (H&E) staining, and pathological sections were made.

After fasting and water deprivation for 4 h, eye blood was collected from each group of experimental mice using the orbital blood collection method. After standing for 2 h, the eyeball blood was centrifuged for 15 min, at 3000 rpm, at 4 °C. The supernatant was taken for packaging, and the specimen was stored at −20 °C or −80 °C before being tested. To induce anesthesia, the experimental mice were place into an anesthesia tank connected to an anesthesia machine. The induction anesthesia concentration of isoflurane is usually adjusted to 4–5%, and the oxygen flow rate is controlled at 1 L/min. The mice faint after 2–3 min. To maintain anesthesia, after inducing anesthesia, the experimental mice were covered with a mask, and made to continuously inhale isoflurane. The concentration of isoflurane was controlled at 2–3%, and the flow rate was controlled at 0.2–0.4 L/min.

### 2.7. Hypoglycemic Effect

The db/db diabetic mouse model was used for this experiment. The mice had an initial blood glucose level of approximately 7 mmol/L and a weight of around 25 g. Their normal diet was replaced with a high-fat diet, and their blood glucose was measured every other day. After two weeks of continuous feeding, mice with blood glucose levels ≥ 11.1 mmol/L were considered successfully models for diabetes. The mice were then randomly divided into five groups (*n* = 6 per group): a CM ICP NPs group (30 mg/kg Cur and 300 mg/kg Met), a mixed Cur–Met NPs group (30 mg/kg Cur and 300 mg/kg Met), a Cur NPs group (30 mg/kg Cur), a Met NPs group (300 mg/kg Met), and a Saline group. Starting from the following day, the mice were intraperitoneally injected with the corresponding treatments for a duration of 7 weeks. The mice were then monitored daily for behavioral habits and mental state, including conditions of insanity, messy hair, difficulty breathing, cachexia, astasia, fighting, and coma. Their body weight and blood glucose levels were recorded weekly. Blood glucose levels were measured using a blood glucose meter (Yuyue, Shanghai, China) with tail vein blood samples. At the end of the therapeutic period, blood samples were collected via orbital sampling to assess glycated serum protein (GSP), insulin resistance index (HOMA-IR), free fatty acids (NEFA), blood lipids (cholesterol [CHO], low-density lipoprotein cholesterol [LDL-C], and high-density lipoprotein cholesterol [HDL-C], as well as key indicators of liver function (ALT, AST, TBIL) and kidney function (CREA, BUN, UA). Finally, the liver, kidney, and pancreas were harvested for H&E staining analysis (*n* = 3 per group). The methods of environmental adaptation, eyeball blood collection, serum procession, and anesthesia for mice are as described above in Section 2.6.3.

### 2.8. Statistical Analysis

All the data were exhibited as the average plus or minus the standard deviation (mean ± SD). Upon juxtaposing the experimental groups, it was revealed through the utilization of Student’s *t*-test or one-way analysis of variance that the results exhibited statistical significance if *p* values were less than 0.05. Statistical analyses were performed using the SPSS 22.0 software. A data graph was drawn using Origin 2018.

## 3. Results and Discussion

### 3.1. Chemical Structure of CM ICP NPs

**Figure 2:**
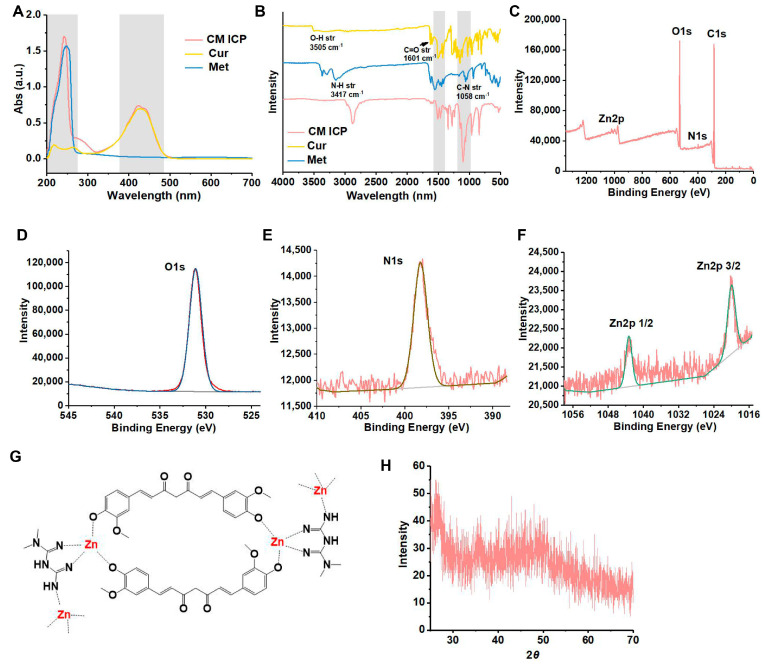
UV-Vis (**A**) and FTIR (**B**) of CM ICPs, Cur, and Met; XPS full spectrum (**C**), XPS high-resolution spectrum (O 1s, (**D**); N 1s, (**E**); Zn 2p, (**F**)), structure (**G**), and XRD spectrum (**H**) of CM ICPs.(The red line represents the original data, while the other colored lines represent the peak fitted data in (**E**,**F**)).

As Figure 2A shows, the results from the UV-Vis analysis indicate that Met exhibits a characteristic absorption peak at 249 nm, while Cur displays peaks at 217 nm, 264 nm, and 427 nm. Additionally, CM ICPs show characteristic peaks at 240 nm and 424 nm, with peak separation near 269 nm. These peak positions are similar to those of Met and Cur, albeit slightly shifted due to coordination, which also causes a superposition of peak shapes. As shown in Figure 2B, the infrared spectrum of Cur reveals an O-H stretching vibration peak at 3505 cm^−1^, a C=O peak at 1601 cm^−1^, a C=C peak at 1523 cm^−1^, and a C-O peak at 1150 cm^−1^. In contrast, Met exhibits an N–H stretching vibration peak at 3147 cm^−1^, a C=N peak at 1546 cm^−1^, and a C–N peak at 1058 cm^−1^. The CM ICPs synthesized from these two materials display a C=C stretching vibration peak at 1340 cm^−1^, corresponding to curcumin, and a C–N peak at 1086 cm^−1^, characteristic of metformin. Notably, the C=C peak of raw curcumin and the C–N peak of raw metformin undergo a blue shift and a red shift, respectively, in CM ICPs. This suggests that these shifts may be attributed to the formation of a Zn coordination bond. These findings suggest that CM ICPs incorporate both Cur and Met. The XPS full spectrum and high-resolution spectra of CM ICPs (Figure 2C–F) reveal binding energy characteristic peaks for C 1s (284.0 eV), N 1s (401.09 eV), O 1s (531.0 eV), and Zn 2p (1019.73 eV), confirming the presence of carbon, nitrogen, oxygen, and zinc in the sample. The binding energies of these elements are detailed in Table S1 and Figure S1. Notably, the binding energy of O 1s in CM ICPs (532.80 eV) is higher than that in pre-coordination Cur (531.70 eV), and the binding energy of N 1s in CM ICPs (398.74 eV) is also higher than in pre-coordination Cur (397.56 eV). The Zn 2p spectrum of C_10_H_14_ZnO_4_ displays two spin-orbitals: 2p3/2 (1019.90 eV) and 2p1/2 (1042.80 eV), corresponding to U and V, respectively. In the XPS spectrum of CM ICPs, the Zn 2p binding energy is split into two peaks: 1020.80 eV and 1043.83 eV. Based on these XPS results and covalent bond theory, it can be inferred that Zn (II) likely accepts lone pairs of electrons from O and N in the coordinated drug molecular chain (Figure 2G). Consequently, the electron transfer behavior during the coordination process is N → Zn and O → Zn, leading to the formation of CM ICPs through coordination.

The preparation of CM ICPs was achieved using the solvent-induced precipitation method, which, due to its inability to crystallize slowly, results in the formation of an amorphous structure distinctly different from MOF crystals. The absence of prominent peaks in the XRD pattern (Figure 2H) further confirms that CM ICPs are amorphous supramolecular polymers with high structural flexibility. This is consistent with the structure of ICPs in paper [25]. Consequently, CM ICPs with infinite coordination structure characteristics were successfully synthesized from Cur, Met, and Zn(C_5_H_7_O_2_)_2_, effectively achieving the encapsulation and binding of Cur and Met. This is owing to the fact that CM ICP NPs are formed by directly coordinating Cur and Met with Zn^2+^ without the use of any carrier material, thus achieving a high encapsulation rate of 100%.

### 3.2. Morphology and Release of CM ICP NPs

**Figure 3:**
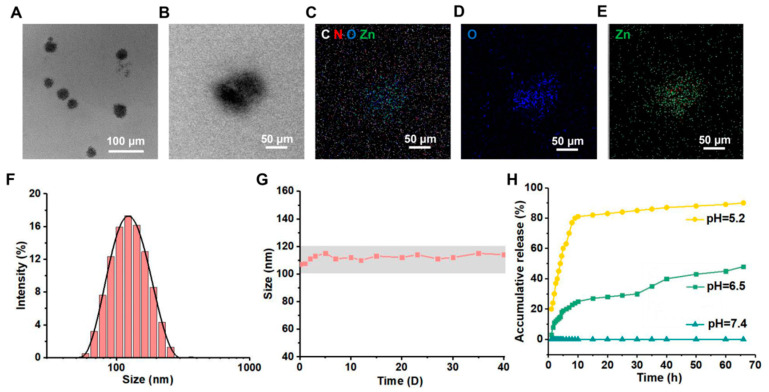
TEM (**A**) and EDS (**B**); (**C**): C, N, O, Zn; (**D**): O; (**E**): Zn; particle size distribution (**F**), stability (**G**), The gray area is between the maximum and minimum values of particle size.), and in vivo drug release (**H**) of CM ICP NPs.

The particle size of nanomedicines is critical for drug delivery and absorption in vivo [37]. Figure 3A–E illustrate the morphology, particle size, and elemental distribution of CM ICP NPs. The findings indicate that CM ICP NPs are uniformly dispersed within the system, forming aggregates in the core of the nanoparticles, with an average particle size of approximately 52 nm. The C, O, and N elements from Cur and Met, along with the coordinated metal Zn^2^⁺, are densely and uniformly distributed throughout the nanoparticles, confirming the successful encapsulation of Cur, Met, and Zn. In Figure 3F, the addition of Pluronic F127 surfactant during the preparation process resulted in the formation of extensive hydration layers in the liquid. Therefore, the hydration particle size measured by DLS (107 ± 20.24 nm) was larger than that observed by TEM. The particle size of CM ICP NPs is conducive to their circulation within the body and absorption by cells [38]. Met primarily exists as a hydrophilic cation at physiological pH and has low fat solubility, making it unlikely for the drug to rapidly and passively diffuse through cell membranes [39,40]. Additionally, the zeta potential of CM ICP NPs is neutral, which is advantageous for in vivo absorption and metabolism, as well as for maintaining stability in vitro [41]. Furthermore, Figure 3G shows that after 40 days of storage at 4.0 °C, there was no significant change in the particle size and drug loading of CM ICP NPs, which remained in the range of 106–116 nm, indicating excellent storage stability in vitro. Met accumulates in the intestines, which makes it difficult for a large proportion of patients to tolerate sufficient amounts. Up to 25% of patients suffer from gastrointestinal side effects related to Met, and about 5% of patients simply cannot tolerate Met [42]. There is an effective method for controlling drug metabolism and improving bioavailability through the controlled release of drugs, which is based on changes in environmental pH [43]. To mimic the acidity and alkalinity in different environments of the body, the drug release characteristics in vivo of CM ICP NPs were investigated. The results are shown in Figure 3H. When pH = 6.5, the CM ICP NPs rapidly release 25% within 10 h, then slowly release, and the release amount gradually increases to around 45% after 30 h. This indicates that the CM ICP NPs can remain in the intestinal fluid for an extended period of time [44]. This can avoid adverse gastrointestinal reactions caused by the accumulation of Met in the intestine. At a pH of 5.2, 80% of the nanoparticles are rapidly released within 10 h, suggesting that they can be effectively released within cells. In an acidic environment, Cur can be rapidly released from CM ICP NPs, but as the pH increases to 7.4, the rate of drug release significantly decreases, and almost no drug release is detected. This indicates that CM ICP NPs have excellent stability in the intraperitoneal pathway and blood circulation. This is because ICP is more sensitive to acidic conditions due to the coordination binding between metals and organic compounds. Therefore, the CM ICP NPs can achieve controlled drug release in vivo.

### 3.3. In Vitro Toxicity of CM ICP NPs

**Figure 4:**
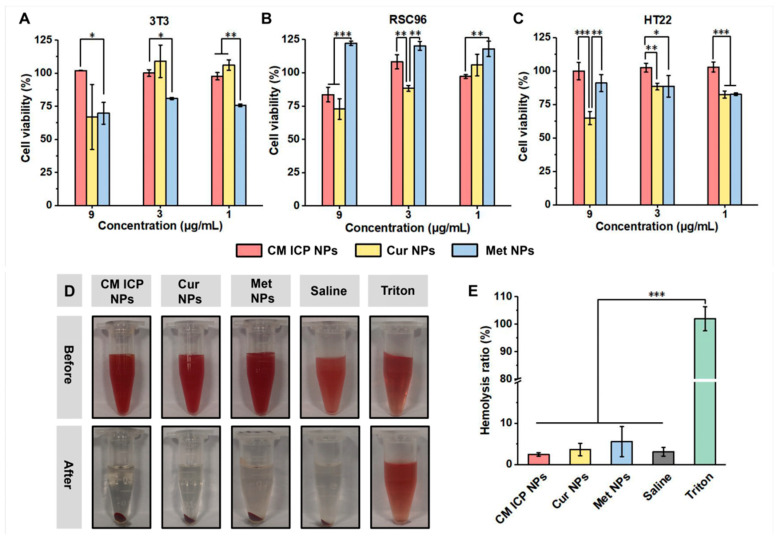
The inhibitory effects of CM ICP NPs, Cur NPs, and Met NPs on three cell lines (3T3) (**A**); RSC96 (**B**); and HT22 (**C**). CM ICP NPs, Cur NPs, and Met NPs were added at concentrations of 1 μg/mL, 3 μg/mL, and 9 μg/mL in vitro; picture (**D**) and numerical (**E**) of hemolysis test results for CM ICP NPs, Cur NPs, Met NPs, Saline, and Triton (they are 15% of the total solution volume). * *p* < 0.05; ** *p* < 0.01; *** *p* < 0.001.

Toxicity testing is a critical prerequisite for ensuring the safe application of drugs. Fibroblasts are widely distributed and abundant in organisms, with advantages such as rapid division and proliferation. Mouse embryonic fibroblasts (3T3) are multipotent mesenchymal cells that can differentiate into different tissues in various culture environments. Therefore, in this paper, 3T3 cells were used to investigate the effects of CM ICP NPs on embryonic toxicity, developmental toxicity, and overall cytotoxicity in diabetic mice. Additionally, two neural cell lines, Schwann cells (RSC96) and mouse hippocampal neurons (HT22), were included to examine the neurotoxicity from different perspectives [45,46,47]. In this study, these three cell lines were employed to assess the in vitro toxicity of CM ICP NPs. As shown in Figure 4A–C, Cur exhibits a slight inhibitory effect on the proliferation of HT22 and RSC96 cells at low concentrations. At a concentration of 9 μg/mL, Cur significantly inhibits the proliferation of all three cells. This could be attributed to Cur inhibiting cell proliferation by suppressing the MAPK/ERK signaling pathway [16]. Met functions by inhibiting mitochondrial respiration [48]. The proliferation rates of both 3T3 and HT22 cell lines were consistently below 100% at all concentrations of Met. However, CM ICP NPs have a minimal impact on cell proliferation within this range, only slightly affecting the viability of RSC96 cells at 9 μg/mL (the proliferation rate is 83.60 ± 5.48%). A cell proliferation rate greater than 100% means that the drug is non-toxic [49]. This suggests that CM ICP NPs are non-cytotoxic at the applied dose. CM ICPs reduce the dosage of Cur and Met, while affecting their coordination and integration structure with cells, diminishing their cytotoxicity. The interaction between CM ICP NPs and cells may have enhanced the cytocompatibility of the compounds due to the influence on the coordination and integration structure of Cur and Met. Table S2 shows that the combination index (CI) of CM ICP NPs is 0.43. The significant synergistic effect between Cur and Met in CM ICP NPs was further confirmed. In the hemolysis assay (Figure 4D,E), the hemolysis rate of the positive control group was 100%, whereas CM ICP NPs and their primary components, Cur NPs and Met NPs, did not cause significant red blood cell lysis. This indicates that CM ICP NPs are safe for intravenous administration.

### 3.4. In Vivo Toxicity of CM ICP NPs

The in vivo acute toxicity of CM ICP NPs was evaluated using C57 mice. Cur and Met are known for their ability to enhance insulin sensitivity, promote glucose utilization, and regulate fat metabolism [50]. Over a 14-day period, two mice in the mixed Cur–Met NPs group exhibited adverse symptoms of cachexia, and five mice died. In contrast, neither the CM ICP NPs group nor the Saline group showed these adverse symptoms or experienced any fatalities (Figure S2 and Table S3). This suggests that CM ICP NPs effectively load and release Cur and Met in a controlled manner, mitigating the risk of uncontrollable, wasteful, and harmful side effects that may arise from directly mixing the drugs.

As shown in Figure S3, there was no significant change in the body weight of mice in any group from 0 to 3 days post injection. However, beginning on day 5, the weight of the mice in the Saline group increased significantly compared to the CM ICP NPs and mixed Cur–Met NPs groups. The body weight of the CM ICP NPs group remained relatively stable, while the mixed Cur–Met NPs group showed a downward trend in weight. These results indicate that CM ICP NPs slowed down weight gain in mice without causing weight loss, suggesting a milder effect on the animals.

**Figure 5:**
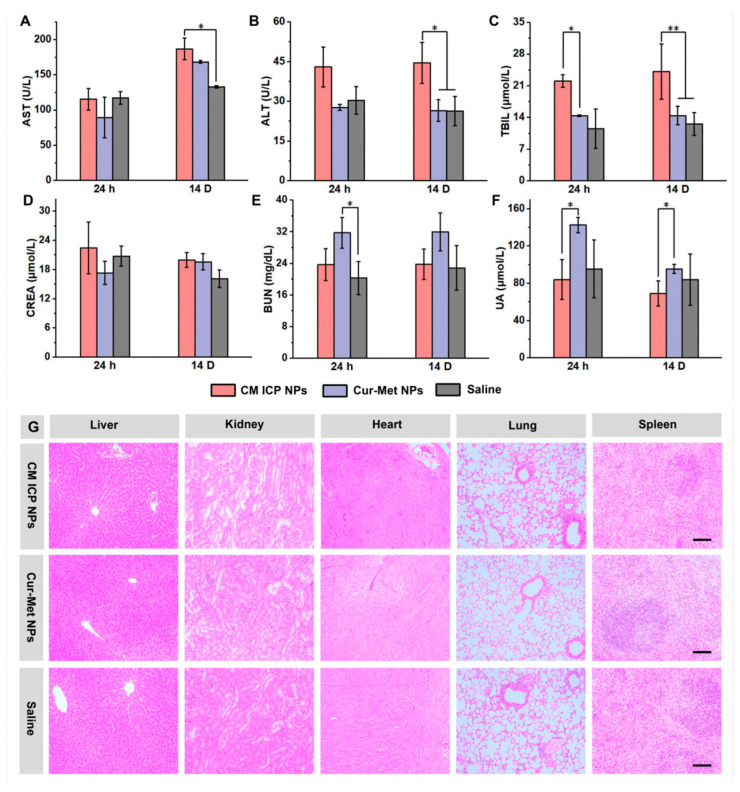
The main biochemical indicators of liver function (AST (**A**); ALT (**B**); TBIL (**C**)) and kidney function (CREA (**D**); BUN (**E**); UA (**F**)) of mice treated with CM ICP NPs, Cur-Met NPs, and saline at 24 h and 14 d (30 mg/kg for Cur and 300 mg/kg for Met). The pathological effects of heart, liver, kidney, spleen, and lungs (**G**) in mice treated with CM ICP NPs, Cur–Met NPs, and saline at 14 d. The dimensions of the scale bar is 100 μm. * *p* < 0.05; ** *p* < 0.01.

Aspartate aminotransferase (AST), alanine aminotransferase (ALT), and total bilirubin (TBIL) are important indicators reflecting liver function. As shown in Figure 5A–C, for mice injected with CM ICP NPs, mixed Cur–Met NPs, and saline separately, it was shown that the levels of AST in the mice increased on day 14 compared to 24 h post injection. There were no significant differences in ALT and TBIL levels between 24 h and 14 days post injection. Meanwhile, the mice injected with CM ICP NPs had higher levels of these indicators than the mixed Cur–Met NPs group and the Saline group. However, the main indicators of liver function in each group remained within the normal range, with no significant pathological changes observed in liver tissue (Figure 5G). This indicates that CM ICP NPs do not cause significant liver damage during metabolism. Kidney function markers such as creatinine (CREA), an indicator of kidney excretion function, showed no significant changes across the groups within the 14-day period, and remained within the normal range (Figure 5D). Although CREA levels only rise significantly when more than 70% of kidney function is compromised [51], other kidney function indicators, such as blood urea nitrogen (BUN) and uric acid (UA), provide additional insights. As shown in Figure 5E,F, BUN levels in mice injected with mixed Cur–Met NPs were significantly higher than those injected with saline, and reached the critically high value of the normal range. The UA levels initially increased and then decreased over time. Pathological analysis revealed lymphocyte infiltration in the kidneys of the mixed Cur–Met group (Figure 5G). In contrast, the UA value of the CM-ICP NPs group was lower than that of the mixed Cur–Met group. The indicators in the CM ICP NPs group were similar to those of the control group, and remained within the normal range. No significant pathological changes were observed in the major organs, including the heart, kidneys, lungs, and spleen (Figure 5G). These findings suggest that the mixed Cur–Met drug injection may cause mild kidney damage during metabolism, while the CM ICP NPs, formed through coordination binding, reduce this damage, and do not harm the major organs during the metabolic process.

### 3.5. Hypoglycemic Performance of CM ICP NPs

**Figure 6:**
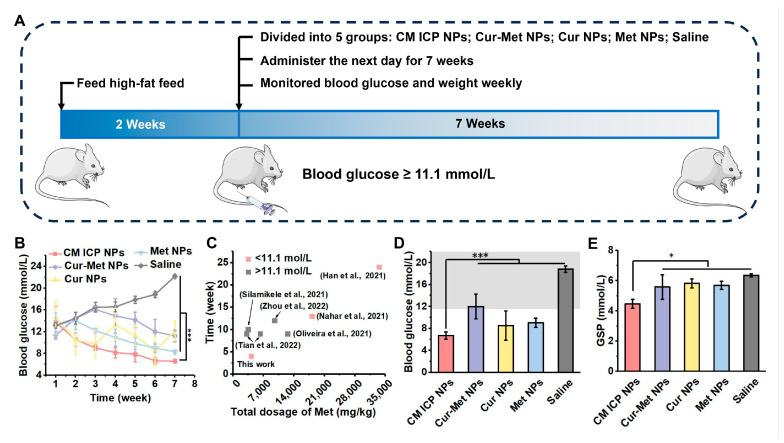
Experimental design of mice (**A**), effects on continuous blood glucose (**B**), The dosage and treatment duration of Met (**C**) [11,40,53,54,55,56], blood glucose at seventh week (**D**), The gray area represents the blood glucose level greater than 11.1 mmol/L), and GSP (**E**) in diabetic model mice of CM ICP NPs, Cur-Met NPs, Cur NPs, Met NPs, and Saline group (30 mg/kg for Cur and 300 mg/kg for Met); * *p* < 0.05; *** *p* < 0.001.

Persistent hyperglycemia in diabetes patients can lead to various organ damages and complications. The effectiveness of blood glucose control is the primary indicator for evaluating hypoglycemic drugs [47]. Therefore, we tested the random blood glucose levels of each group of mice; the experimental mouse design is shown in Figure 6A, and the results are shown in Table S4 and Figure 6B–E. During the experiment, no adverse conditions were observed in any of the groups of mice. Due to the absence of drug interference, the blood glucose levels of the Saline group of mice remained elevated. The blood glucose levels of the Cur NPs group of mice showed a trend of initial decrease, then increase, and then decrease again. This indicates that although Cur NPs can quickly reduce blood glucose in mice, it may rebound in a short period of time, making their hypoglycemic effect unstable. This is due to the fast metabolism, low absorption, and low bioavailability of Cur NPs when used alone in an organism [52]. In Figure 6B, the blood glucose levels of mice injected with Met NPs steadily decreased from the third week onwards, and dropped below 11.1 mmol/L by the fourth week (the cumulative dosage of Met injected is 4200 mg/kg). In most research works, Met intake is carried out through gastric irrigation, enema, or drinking water. When using similar amounts of Met, even if the treatment time is prolonged, the blood glucose levels of mice cannot be reduced to a safe range. For example (see Figure 6C), Silamiķele et al. [53] dissolved Met in drinking water and fed mice for ten weeks. After that, the blood glucose levels of the mice remained at 15.01 mmol/L (the cumulative dosage of Met injected is 3500 mg/kg). Similarly, Tian et al. [54]. found that the blood glucose levels of mice remained above 11.1 mmol/L (the cumulative dosage of Met injected is 6300 mg/kg). In order to achieve therapeutic effects, the dosage and treatment time of Met were increased. For instance, Nahar et al. [55] used a dosage of 18,200 mg/kg and administered gastric irrigation continuously for 13 weeks, while Han et al. [56] even administered gastric irrigation continuously for 24 weeks to mice (the cumulative dosage of Met injected is 33,600 mg/kg). Therefore, intraperitoneal injection can effectively reduce the dosage of Met and achieve ideal therapeutic effects. However, the mixed Cur–Met NPs group of mice showed a continuous increase in blood glucose levels, before a gradually decrease after 3 weeks, but their blood glucose levels remained significantly higher than those of the other groups. This suggests that the pure mixed administration of Cur–Met NPs not only fails to exhibit synergistic effects, but may even lead to a phenomenon of 1 + 1 < 2, due to the mutual interference of the action pathways. It is worth noting that the blood glucose levels of mice treated with CM ICP NPs decreased to below 11 mmol/L in the second week, and then continued to decrease over time, reaching 6.7 ± 0.65 mmol/L by 7 weeks. Additionally, the GSP indicators, which reflect long-term blood glucose control levels, also showed that the CM ICP NPs group had significantly lower levels than the other groups (Figure 6D). This demonstrates that the ICP nanoparticles formed by Cur and Met can effectively solve the problem of pathway interference caused by the simple drug mixture, improve its utilization rate, regulate the hypoglycemic action mode of both drugs in diabetic mice, and can achieve excellent hypoglycemic and glucose control effects. Furthermore, CM ICP NPs can also slow down the weight growth rate of diabetic mice (Figure 6E). The unique physicochemical properties of nanoparticles (NPs) confer significant advantages in terms of biocompatibility, target specificity, cellular uptake, and bioavailability. This provides a promising strategy for combined treatment with diabetes drugs [57].

### 3.6. Mechanism of Action of CM ICP NPs

#### 3.6.1. Effects of CM ICP NPs on Insulin Resistance

**Figure 7:**
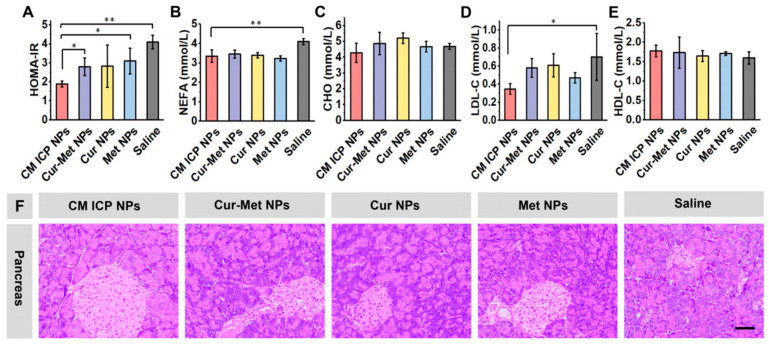
HOMA-IR (**A**), NEFA (**B**), CHO (**C**), LDL-C (**D**), HDL-C (**E**), and pancreatic tissue (**F**) in diabetic model mice of CM ICP NPs, Cur–Met NPs, Cur NPs, Met NPs, and Saline group (30 mg/kg for Cur and 300 mg/kg for Met). The dimensions of the scale bar is 100 μm. * *p* < 0.05; ** *p* < 0.01.

Diabetes is a metabolic disease caused by insufficient insulin secretion or utilization. Insulin plays a crucial role in promoting glucose utilization, which is converted into glycogen and stored in the body for energy. When insulin utilization decreases and cannot effectively control blood sugar, this results in insulin resistance. This condition is usually asymptomatic, and is often diagnosed through insulin index [50,58]. In Figure 7A, the insulin resistance values of the CM ICP NPs group have returned to normal levels, while the other groups still show varying degrees of insulin resistance. Compared with the normal pancreatic tissue (Figure S5), the H&E staining results of the pancreas in Figure 7F reveal that the Saline group has significantly reduced islet numbers, atrophied islets with a loose and irregular structure, and disordered cell arrangement. In comparison, the islet morphology in each treatment group has significantly improved. The CM ICP NPs group shows round and regular islets with clear boundaries, and a tight and neat cell arrangement. Wang et al. [59] found that improving insulin resistance can be beneficial in treating type 2 diabetes mellitus (T2DM). CM ICP NPs effectively improve pancreatic islet atrophy and structural lesions, significantly reduce systemic insulin resistance in mice, and restore normal glucose regulation.

Met and Cur can improve insulin resistance by promoting the recovery of β-cell function, inhibiting liver glucose production, and increasing peripheral tissue utilization of glucose. Metformin achieves this effect through direct and indirect effects on mediators from the initial stages of the insulin signaling pathway, AMPK activation, GLUT4 trafficking and translocation mediators, and complex AMPK-dependent and -independent epigenetic modifications [60]. The action mechanism of Cur may increase pancreatic islet activity by inhibiting poly ADP-ribose polymerase-1 activation, as well as increasing the number of small islets by enhancing HSP-70 and HO-1 [61]. These two act from different pathways and targets. It is obvious that simple drug mixing provides little help in terms of complementing or synergizing with each other. However, the CM ICP NPs showed lower insulin resistance. This may be due to the coordination and combination changes in their pharmacokinetics, which enhance the synergistic effect.

#### 3.6.2. Effects of CM ICP NPs on Lipid Metabolism

Free fatty acids (NEFA) are lipids present in the human body, and are substances broken down from neutral fats. NEFA can affect glucose metabolism through the glucose fatty acid cycle [62]. Increase in NEFA is a common lipid metabolism disorder in T2DM, which can inhibit the uptake and utilization of glucose in body tissues and provide raw materials for gluconeogenesis. From Figure 7B, it can be observed that the NEFA of the CM ICP NPs group is lower than that of the Saline group. CM ICP NPs can improve lipid metabolism in mice. Therefore, the blood lipids of each group of mice were further examined, and the results are shown in Figure 7C–E. CHO refers to the total amount of cholesterol carried by all the lipoproteins in the blood. Although there is no significant difference between the groups of mice, they are all higher than the normal range, indicating a certain degree of lipid metabolism disorder in all the groups of mice. However, it is worth noting that the LDL-C of the CM ICP NPs group is lower than that of the other groups, and the HDL-C is higher than that of the Saline group. This shows that CM ICP NPs can improve lipid metabolism disorder to a certain extent, and reduce the risk of atherosclerotic angiopathy and other diseases in diabetic mice.

#### 3.6.3. Effects of CM ICP NPs on the Liver

**Figure 8:**
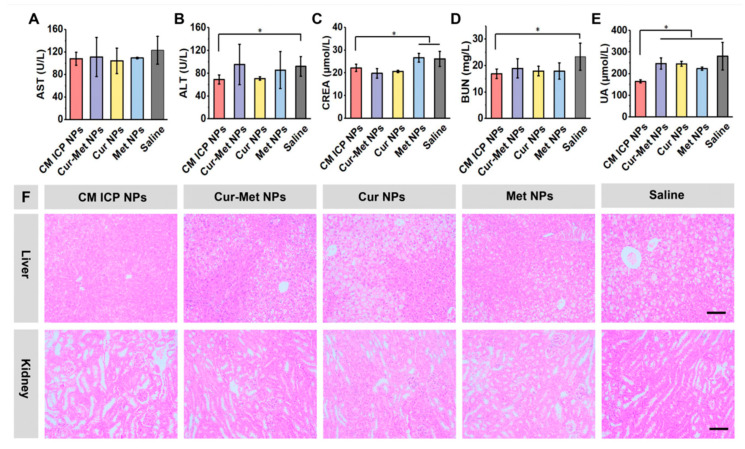
AST (**A**), ALT (**B**),CREA (**C**), BUN (**D**), UA (**E**), and liver and kidney tissue (**F**) in diabetic model mice of the CM ICP NPs, Cur–Met NPs, Cur NPs, Met NPs, and Saline groups (30 mg/kg for Cur and 300 mg/kg for Met). The dimensions of the scale bar is 100 μm. * *p* < 0.05.

Both sugar metabolism and lipid metabolism are inseparable from the metabolic effects of the liver and kidneys. Therefore, the characteristic indicators of liver and kidney function, as well as histopathology of each group, were analyzed. From Figure 8A,B, it was found that the mean values of AST in the CM ICP NPs group, mixed Cur–Met NPs group, Cur NPs group, and Met NPs group were all lower than those in the Saline group, but there was no significant difference among the groups. The ALT levels in the CM ICP NPs group were significantly lower than those in the Saline group, (92.20 ± 17.23, near the critical high value). The liver tissue slices of the Saline group in Figure 5G show the normal image of a liver with H&E staining. In Figure 8F, the Saline group showed significant liver tissue lesions with severe steatosis, abundant lipid vacuoles in the cytoplasm, and swollen and disorderly arrangement of liver cells. After administration, some liver cell morphology in the Cur NPs group, Met NPs group, and mixed Cur–Met NPs group returned to normal. The liver cell swelling and fat vacuoles were partially improved. The liver cell morphology in the CM ICP NPs group basically returned to normal, with only a small number of liver cells exhibiting lipid degeneration and disordered arrangement. The above results indicate that other groups had improved liver cell steatosis, but not significantly, while the CM ICP NPs group had a significantly better improvement in liver cell steatosis than the other groups, and experienced a certain regulatory effect on their renal metabolism.

#### 3.6.4. Effects of CM ICP NPs on the Kidneys

The CREA of the CM ICP NPs group was lower than that of the Met NPs group and Saline group, but higher than that of the mixed Cur–Met NPs group and Cur NPs group (Figure 8C). Their BUN and UA were significantly lower than other groups (Figure 8D,E). The H&E stained sections of normal mouse kidney tissue (Figure 5G) were used as positive controls. The results of kidney pathological sections (Figure 8F) show that in the Saline group, the number of glomeruli decreased, the structure was scattered and irregular, and there was tearing of the cystic cavity of Baumann’s with a small amount of inflammatory cell infiltration; there was distortion of the renal tubular morphology accompanied by swelling and hypertrophy, and compression of the official cavity; and they had a blurred renal interstitium. Compared with the Saline group, the number of glomeruli in each treatment group increased to varying degrees, and the degree of renal lesions improved. The CM ICP NPs group showed the most significant therapeutic effect, with regular glomerular morphology and almost no inflammatory cell infiltration in the renal tissue. The morphology of the Baumann’s capsule was regular, and the renal tubular lumen was normal. This indicates that the improvement of renal lesions in the CM ICP NPs group was significantly better than that in other groups.

Met does not undergo changes in the body, and remains unchanged when secreted through rapid kidney excretion [61]. Impaired kidney function can slow down elimination and may lead to accumulation of Met [63]. Curcumin can restore renal integrity by increasing blood urea nitrogen, decreasing levels of albuminuria and enzymuria, and normalizing glutathione [64,65,66]. These mechanisms may be due to Cur-mediated activation of AMP, which reduces the expression of VEGF and VEGF receptor, diminishes the activities of PKC-α and PKC-β, and suppresses sterol regulatory element-binding protein (SREBP)-1c [67,68]. Clinical trials have further shown that Cur is beneficial for the treatment of end-stage renal disease [69]. ICP technology enhances the efficacy of Cur, which has a certain protective effect on the kidneys of T2DM mice.

To sum up, CM ICP NPs can regulate insulin resistance and improve lipid metabolism and glucose metabolism though the synergistic effects of multiple paths. They have a certain effect on the treatment of diabetic mice by improving the pancreas, liver, kidney and other tissues.

## 4. Conclusions

In order to improve the diabetic therapeutic efficiency of Met, a tightly bound infinite coordination polymer, CM ICP NP, was prepared by coordinating Met and Cur with Zn^2+^. These nanoparticles exhibit a high drug encapsulation rate (100%) and stable dispersion in aqueous systems, with a controlled release profile in acidic environments, and a particle size of 107 ± 20.24 nm. The chemical structure and coordination state of the nanoparticles were characterized using FTIR spectroscopy, XPS, and XRD. Toxicity assessments in both cell and mouse models confirmed the absence of in vivo and in vitro toxicity. Diabetic mice treated with intraperitoneal injections of CM ICP NPs demonstrated significantly improved glucose control, with blood glucose levels reduced to 6.7 ± 0.65 mmol/L by the seventh week, markedly lower than in those treated with a mixture of the drugs or saline. The therapeutic mechanism appears to involve the restoration of normal glucose regulation in diabetic mice by improving insulin resistance, lipid metabolism, and liver and kidney function under the synergistic effects of Cur and Met. This study provides a promising approach for the combination therapy of diabetes using dual-drug coordination polymer nanoparticles. Further research is needed to explore the multiple paths mechanism of the synergistic effect, to provide theoretical support for drug development.

## Data Availability

The original contributions presented in the study are included in the article/supplementary material, further inquiries can be directed to the corresponding authors.

## Supplementary Materials

The following supporting information can be downloaded at https://www.mdpi.com/article/10.3390/jfb15120388/s1. Table S1: Electron binding energies of key elements in CM ICP and Cur, Met, Zn(C_5_H_7_O_2_)_2_. Table S2: The IC50 value of CM ICP NPs; Table S3: Mice status treated with CM ICP NPs, mixed Cur-Met NPs, and Saline after 14 days; Table S4: Mice status treated with CM ICP NPs, mixed Cur-Met NPs, Cur NPs, Met NPs, and Saline after 7 weeks; Figure S1: The O 1s XPS fine spectrum of Cur (A); The N 1s XPS fine spectrum of Met (B); The Zn 2p XPS fine spectrum of Zn(C_5_H_7_O_2_)_2_ (C); Figure S2: The survival rate of mice treated with CM ICP NPs, mixed Cur-Met NPs, and Saline; Figure S3: The weight of mice treated with CM ICP NPs, mixed Cur-Met NPs, and Saline. ** *p* < 0.01; *** *p* < 0.001; Figure S4: The body weight in diabetes model mice of CM ICP NPs and Saline group. ** *p* < 0.01; *** *p* < 0.001; Figure S5: The image of pancreatic tissue stained with hematoxylin and eosin in normal db/db mouse [58].
