# Supplementary material for: Curcumin and Metformin Infinite Coordination Polymer Nanoparticles for Combined Therapy of Diabetic Mice via Intraperitoneal Injections
Source: J Funct Biomater. 2024 Dec 21;15(12):388. doi: 10.3390/jfb15120388 (PMC11677912; doi:10.3390/jfb15120388)
Supplement: Supplementary file 1 [file jfb-15-00388-s001.zip › jfb-3331688-supplementary.pdf]

Supplementary Information

Curcumin and Metformin Infinite Coordination Polymer Nanoparticles for Combined Therapy of Diabetic Mice via Intraperitoneal Injections

Siwei Sun [1,2], Xinyi Hou [2,3], Ke Li [1,2], Chenqi Huang [4], Yu Rong [1,2], Jiao Bi [1,2], Xueping Li [2,5,*], Daocheng Wu [4,*]

[1] Institute of Basic and Translational Medicine, Xi'an Medical University, Xi'an 710021, China

[2] Xi'an Key Laboratory for Prevention and Treatment of Common Aging Diseases, Translational and Research Centre for Prevention and Therapy of Chronic Disease, Xi'an Medical University, Xi'an 710021, China

[3] School of Pharmacy, Xi'an Medical University, Xi'an 710021, China

[4] Key Laboratory of Biomedical Information Engineering of the Ministry of Education, School of Life Science and Technology, Xi'an Jiaotong University, Xi'an 710049, China

[5] School of Clinical Medicine, Xi'an Medical University, Xi'an 710021, China

* Correspondence: Xueping Li, Email: lxp86@xiyi.edu.cn; Daocheng Wu: Email: wudaocheng@mail.xjtu.edu.cn, orcid.org/0000-0002-6183-539X; 1261

Siwei Sun and Xinyi Hou contributed equally to this work.

1. Preparation method of samples

1.1. Preparation of Cur-Met NPs

Cur (7.28 mg) and Pluronic F127 (14.56 mg) were fully dissolved in 80 μL DMSO. The solution was slowly injected into the bottom of Met (72.8 mg) saline solution (1920 μL) and then stirred evenly. The mixed Cur-Met NPs should be prepared and used as needed.

1.2. Preparation of Cur NPs

Cur (7.28 mg) and Pluronic F127 (14.56 mg) were fully dissolved in 80 μL DMSO. The solution was slowly injected into the bottom of saline solution (1920 μL) and then stirred evenly. Cur NPs should be prepared and used as needed.

1.3. Preparation of Met NPs

Met (72.8 mg) were fully dissolved in 1920 μL saline solution. Met NPs should be prepared and used as needed.

2. Determination of encapsulation rate to Cur and Met

CM ICP NPs were subjected to centrifugation and concentration by the centrifugal dialysis tube with 10 kDa cut-off molecular weight under 3000 r/min and 20 minutes. The supernatant was used to calculate the remaining amounts of Cur and Met by ultraviolet-visible absorption spectroscopy. The calculation formula for encapsulation rate is as following formula (1-1). W_1 and W_2 represent respectively the weight of Met and Cur in CM ICP NPs and in preparation, g.

$$\text{Encapsulation rate (\%)} = \frac{W_1}{W_2} \times 100\% \tag{1-1}$$

3. Quantitative analysis of synergistic effect

In order to quantitatively evaluate the synergistic effect of Cur and Met in CM ICP NPs, a combination index (CI) calculation method was used in this study. The calculation is based on the following formula (1-2) :

$$CI = \frac{D_1}{D_{m1}} + \frac{D_2}{D_{m2}} \tag{1-2}$$

D_1 and D_2 represent the concentration of 50 % cell inhibition effect when the two drugs are used in combination, μM. D_{m1} and D_{m2} represent the concentration required for each drug to achieve the same inhibition effect when used alone, μM. The CI value is less than 1 indicates that there is a synergistic effect between the drugs, that is, the effect of the combination of the two drugs is better than that of the single use. In this experiment, the IC_{50} value was used to quantify the synergistic effect of Cur and Met under different conditions. mouse breast cancer cells (4T-1) were used.

4. Supplementary figures and tables

Table S1 Electron binding energies of key elements in CM ICP and Cur, Met, $Zn(C_5H_7O_2)_2$.

Sample	Bindingenergy[eV]			
	O 1s	N 1s	Zn 2p	
	-		Zn 2p 3/2	Zn 2p 1/2

CM ICP NPs	532.80	398.74	1020.80	1043.83
Cur	531.70	-	-	-
Met	-	397.56	-	-
$C_{10}H_{14}ZnO_4$	-	-	1019.90	1042.80

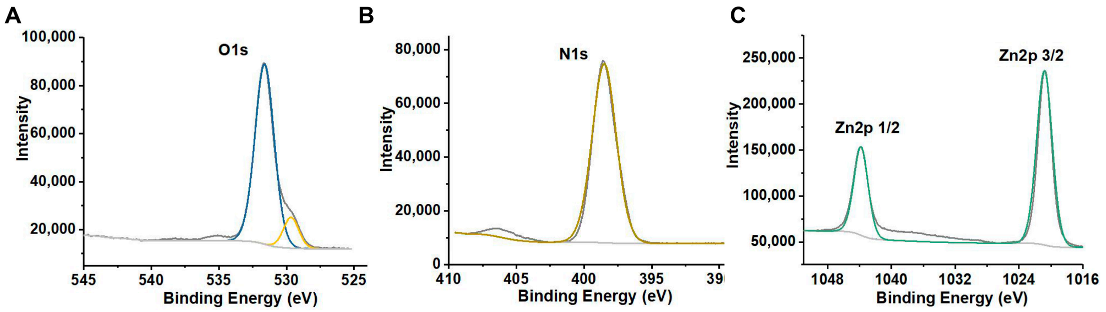

Figure S1 The O 1s XPS fine spectrum of Cur (A); The N 1s XPS fine spectrum of Met (B); The Zn 2p XPS fine spectrum of $Zn(C_5H_7O_2)_2$ (C).

Table S2 The IC_{50} value of CM ICP NPs

Group	IC_{50} (μM)
Free Met	7570
Free Cur	142
CM ICP NPs	81.24

Table S3 Mice status treated with CM ICP NPs, mixed Cur-Met NPs, and Saline after 14 days

Group	CM ICP NPs	Cur-Met NPs	Saline
Insane	non	non	non
Hair messy	non	non	non
Difficult Breathing	non	non	non
Cachexia	non	2	non
Astasia	non	non	non
Fight	non	non	non
Coma	non	non	non
Death	non	5	non

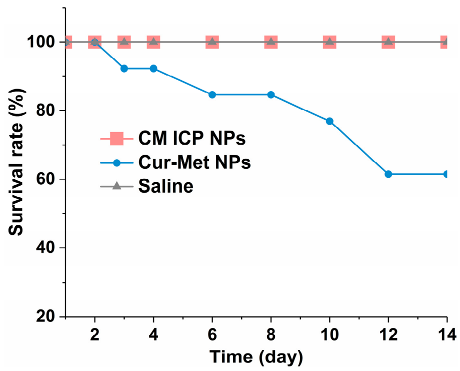

Figure S2 The survival rate of mice treated with CM ICP NPs, mixed Cur-Met NPs, and Saline.

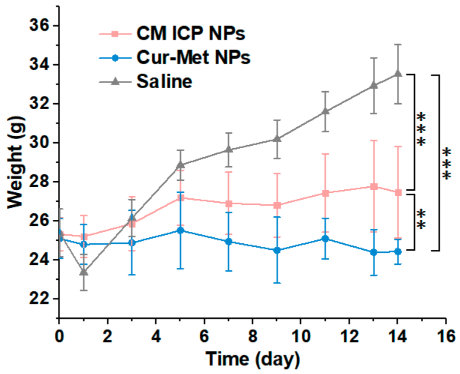

Figure S3 The weight of mice treated with CM ICP NPs, mixed Cur-Met NPs, and Saline.

**$p < 0.01$; ***$p < 0.001$.

Table S4 Mice status treated with CM ICP NPs, mixed Cur-Met NPs, Cur NPs, Met NPs, and Saline after 7 weeks

Group	CM ICP NPs	Cur-Met NPs	Cur NPs	Met NPs	Saline
Insane	non	non	non	non	non
Hair messy	non	non	non	non	non
Difficult	non	non	non	non	non
Cachexia	non	non	non	non	non
Astasia	non	non	non	non	non
Fight	non	non	non	non	non
Coma	non	non	non	non	non
Death	non	non	non	non	non

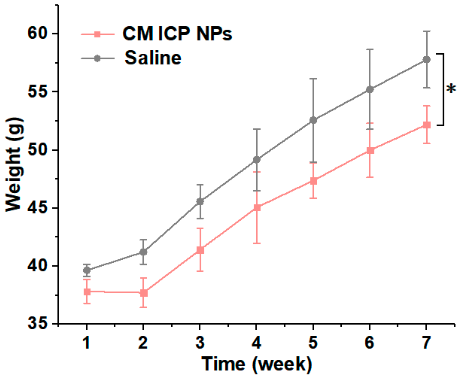

Figure S4 The body weight in diabetes model mice of CM ICP NPs and Saline group.

**p < 0.01; ***p < 0.001.

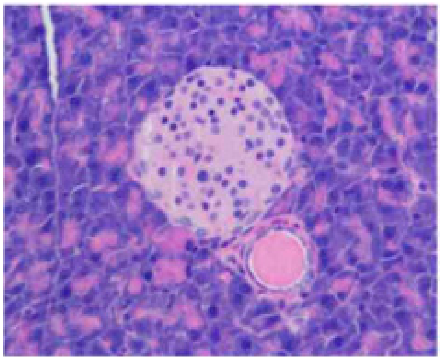

Figure S5 The image of pancreatic tissue stained with hematoxylin and eosin in normal db/db mouse (*Zhou et al., 2021*)

Reference

Zhou, D.Y.; Chen, L.J.; Mou, X. Acarbose ameliorates spontaneous type-2 diabetes in db/db mice by inhibiting PDX-1 methylation. *Mol. Med. Rep.* **2021**, 23, 72.
